# Evaluation of rapid screening and pre-emptive contact isolation for detecting and controlling methicillin-resistant *Staphylococcus aureus *in critical care: an interventional cohort study

**DOI:** 10.1186/cc3982

**Published:** 2006-02-06

**Authors:** Stephan Harbarth, Cristina Masuet-Aumatell, Jacques Schrenzel, Patrice Francois, Christophe Akakpo, Gesuele Renzi, Jerome Pugin, Bara Ricou, Didier Pittet

**Affiliations:** 1 Associate Hospital Epidemiologist, Infection Control Program, Geneva University Hospitals, Geneva, Switzerland; 2 Research Fellow, Infection Control Program, Geneva University Hospitals, Geneva, Switzerland; 3 Director, Clinical Microbiology Laboratory, Geneva University Hospitals, Geneva, Switzerland; 4 Senior Research Associate, Genomic Research Laboratory, Geneva University Hospitals, Geneva, Switzerland; 5 Infection Control Practitioner, Infection Control Program, Geneva University Hospitals, Geneva, Switzerland; 6 Laboratory technician, Clinical Microbiology Laboratory, Geneva University Hospitals, Geneva, Switzerland; 7 Attending, Intensive Care Division, Geneva University Hospitals, Geneva, Switzerland; 8 Director, Infection Control Program, Geneva University Hospitals, Geneva, Switzerland

## Abstract

**Introduction:**

Rapid diagnostic tests may allow early identification of previously unknown methicillin-resistant *Staphylococcus aureus *(MRSA) carriers at intensive care unit (ICU) admission. The aim of this study was twofold: first, to assess whether a new molecular MRSA screening test can substantially decrease the time between ICU admission and identification of MRSA carriers; and, second, to examine the combined effect of rapid testing and pre-emptive contact isolation on MRSA infections.

**Method:**

Since November 2003, patients admitted for longer than 24 hours to two adult ICUs were screened systematically on admission using quick, multiplex immunocapture-coupled PCR (qMRSA). Median time intervals from admission to notification of test results were calculated for a five-month intervention phase (November 2003–March 2004) and compared with a historical control period (April 2003–October 2003) by nonparametric tests. ICU-acquired MRSA infection rates were determined for an extended surveillance period (January 2003 through August 2005) and analyzed by Poisson regression methods.

**Results:**

During the intervention phase, 97% (450/462) of patients admitted to the surgical ICU and 80% (470/591) of patients admitted to the medical ICU were screened. On-admission screening identified the prevalence of MRSA to be 6.7% (71/1053). Without admission screening, 55 previously unknown MRSA carriers would have been missed in both ICUs. Median time from ICU admission to notification of test results decreased from 87 to 21 hours in the surgical ICU (*P *< 0.001) and from 106 to 23 hours in the medical ICU (*P *< 0.001). In the surgical ICU, 1,227 pre-emptive isolation days for 245 MRSA-negative patients were saved by using the qMRSA test. After adjusting for colonization pressure, the systematic on-admission screening and pre-emptive isolation policy was associated with a reduction in medical ICU acquired MRSA infections (relative risk 0.3, 95% confidence interval 0.1–0.7) but had no effect in the surgical ICU (relative risk 1.0, 95% confidence interval 0.6–1.7).

**Conclusion:**

The qMRSA test decreased median time to notification from four days to one day and helped to identify previously unknown MRSA carriers rapidly. A strategy linking the rapid screening test to pre-emptive isolation and cohorting of MRSA patients substantially reduced MRSA cross-infections in the medical but not in the surgical ICU.

## Introduction

Nosocomial infections caused by methicillin-resistant *Staphylococcus aureus *(MRSA) are associated with significant adverse outcomes and increased health care costs [1]. Patients colonized with MRSA serve as a reservoir for spread within the health care environment, mainly through the hands of health care workers. Active surveillance by patient screening and intensive control measures represent attempts to decrease this reservoir, with the ultimate goal of reducing MRSA infection rates [2].

The most efficient approach to control of endemic MRSA remains controversial [3-6]. Several authorities have suggested that screening on admission to intensive care units (ICUs) and subsequent patient isolation may decrease the risk for MRSA cross-infection [7-11]. Rapid screening tests may further improve MRSA control, because traditional microbiological methods for MRSA screening are slow [12]. Delays in receiving screening results means either that negative patients remain isolated for too long or that positive patients remain a hidden reservoir for cross-infection. With the availability of rapid molecular MRSA screening methods, determining their value in daily practice is of great importance.

The purpose of this study, conducted in two adult ICUs with endemic MRSA, was to test the hypothesis that a new molecular technique that enables early detection of MRSA carriage can substantially decrease the time between ICU admission and notification of screening results. In addition, we evaluated the effect of a strategy combining early MRSA detection and pre-emptive contact isolation on the rate of ICU-acquired MRSA infections.

## Materials and methods

### Setting and study populations

The Geneva University Hospital, Switzerland, is a 2,200-bed primary and tertiary medical centre, to which approximately 47,000 patients are admitted annually. The surgical ICU is an 18-bed referral unit, admitting 1,650 patients per year for close observation and treatment after multiple trauma and major surgery for a mean duration of 4.0 days. The medical ICU has 18 beds, 1,700 admissions and an average length of stay of 3.7 days. In 2002 the medical ICU had a nosocomial MRSA acquisition rate of 2.5 new MRSA cases per 1,000 patient days, whereas the surgical ICU had 3.5 new cases per 1,000 patient days.

### Study design and outcomes

This interventional cohort study compared a new molecular technique enabling quick MRSA screening (qMRSA) with a standard method in patients admitted to the two adult ICUs at the Geneva University Hospital. From November 2003, patients admitted for longer than 24 hours were screened systematically on ICU admission using the qMRSA test, which is based on a multiplex immunocapture-coupled PCR, to identify patients colonized with MRSA. Median time interval (in hours) from ICU admission to notification of test results to ICU care givers was the primary end-point, which was determined over a five-month period (November 2003–March 2004; phase II) and compared with a historical control period (April 2003–October 2003; phase I), during which conventional culture techniques were used. In phase I, on-admission screening was performed only in those patients at high risk for MRSA carriage (for example, with previously identified MRSA colonization or transfer from a long-term care facility) [13,14].

ICU-acquired MRSA infection rates were determined for an extended surveillance period (January 2003 through August 2005), which encompassed phase III (April 2004–August 2005). Systematic discharge screening was only introduced in November 2003; therefore, it was not possible to evaluate MRSA acquisition rates for the entire study period. Table 1 summarizes the different phases and interventions in the study.

### Screening procedure

Swabs were taken using a cotton stick moistened with sterile 0.9% saline solution. They were collected from both anterior nares and perineal region in all patients and, if positive, from catheter insertion sites, skin lesions and urine in catheterized patients [15,16]. The study was approved by the institutional review board as a continuous quality improvement project, providing direct benefit to involved patients. Informed consent was therefore not required.

### Microbiologic procedures

#### Conventional technique

Swabs were first streaked onto ORSA plates (Oxacillin Resistance Screening Agar; Oxoid, Basingstoke, UK) and then suspended in 2 ml colistin-salt broth (brain–heart infusion with 10 μg colistin/ml and 2.5% NaCl) as a backup media. Suspect colonies were confirmed by Pastorex agglutination (Bio-Rad, Reinach, Switzerland), positive reaction on DNase agar, and growth on Mueller-Hinton oxacillin agar (6 μg oxacillin/ml). The presence of MRSA was confirmed using the Vitek 2 identification and susceptibility testing cards for Gram-positive bacteria (bioMérieux, Marcy l'Etoile, France).

#### Workflow

Before the study we accelerated the workflow in the microbiology laboratory to minimize turnaround times (TATs) of the conventional method by optimizing laboratory organization and selecting faster methods. Median TATs (time interval between sample delivery to the laboratory and report of the results) decreased subsequently from 101 hours (year 2000) to 73 hours (year 2002).

#### Rapid technique

The quick multiplex immunocapture-coupled quantitative PCR allows for same-day diagnosis of MRSA carriage through detection of the *mecA *gene (either in *S. aureus *or *S. epidermidis*), even in the presence of samples heavily contaminated by methicillin-resistant *S. epidermidis *[17]. Swab samples are resuspended in a buffer, and then *S. aureus *is immunocaptured using monoclonal antibodies coupled to magnetic beads and directed against protein A. After bacterial lysis, the presence of MRSA is assessed using a multiplexed real-time PCR (qMRSA) assay, as previously described [17]. If *mecA *cannot be linked to the presence of methicillin-resistant *S. epidermidis*, then MRSA is reported to be present. qMRSA tests were performed five days per week, excluding weekends.

#### Test characteristics

The diagnostic performance of the qMRSA test in critically ill patients was assessed during a pilot phase before the start of the present study. Results of conventional MRSA culturing procedures, including a backup broth culture, were used as an imperfect reference standard, because PCR-based tests are known to yield more positive results than are cultures [18,19]. With a prevalence of MRSA carriage on admission of 14% (31/219), the sensitivity and specificity of the qMRSA test were 84% and 94%, respectively, compared with conventional procedures, with a corresponding negative predictive value of 97%. Analysis of false-negative qMRSA results (*n *= 5) indicated that they arose from samples where MRSA was recovered from backup broth only.

### Infection control procedures

In both ICUs, MRSA control measures included contact isolation of identified MRSA carriers in rooms with flagged doors and dedicated material (gowns, gloves, masks); spatial separation of patients into cohorts in case of large clusters; topical decolonization (nasal mupirocin ointment and antiseptic body washing of known MRSA carriers for at least five days); a computerized MRSA alert system; and regular feedback of surveillance results [13,15,20].

Because of some earlier clusters of MRSA cross-transmission, since 1999 the surgical ICU has applied a pre-emptive isolation policy in patients at high risk for MRSA carriage on admission (for example, transfer from another health care facility) [21]. In November 2003 (surgical ICU) and April 2004 (medical ICU), the ICUs introduced a general pre-emptive isolation policy for all patients. Patients were presumptively placed under contact precautions until the results of the qMRSA test were found to be negative.

### Data collection and methicillin-resistant *Staphylococcus aureus *surveillance

TATs for MRSA screening and work up were recorded for study phases I and II (weekends and public holidays excluded) with the help of computerized laboratory databases and stored in a log file for the purpose of statistical analysis. Retrieved parameters were the following time intervals (in hours): time from ICU admission to MRSA screening; time from screening to sample delivery to the laboratory; and time from arrival at the laboratory to reporting of results. During phase III, detailed TATs were not recorded.

MRSA infections were monitored by dedicated infection control nurses who visited the ICUs daily (excluding weekends) and performed prospective surveillance of MRSA infections using modified US Centers for Disease Control and Prevention definitions [22]. The nurses screened a wide range of data, gathered from medical records, nursing charts and microbiology reports [23,24].

### Definitions

MRSA infection was considered to be ICU acquired if the patient developed the infection 48 hours after admission to one of the ICUs and had not been colonized or infected with MRSA within the previous week before ICU admission. A previously unknown MRSA case was defined as any patient in whom MRSA was isolated for the first time on ICU admission [16]. A pre-emptive isolation day was defined as a day in which an ICU patient stayed under contact precautions while awaiting the results of the on-admission MRSA screening. The incidence rate of ICU-acquired MRSA infections was defined as the number of newly identified patients with MRSA infection divided by the number of patient days at risk [15].

### Statistical analysis

Time intervals from admission to notification of test results were expressed as medians and compared using nonparametric tests. To compare incidence rates of ICU-acquired MRSA infections over time, Poisson regression analysis was performed because rates were low [15,25]. This analysis included data from January 2003 through August 2005. The number of ICU-acquired MRSA infections in a given month was the dependent variable. Only one MRSA infection was considered per patient per ICU stay. Preintervention or postintervention status (extension of pre-emptive isolation and introduction of the qMRSA test to the surgical ICU; initiation of qMRSA testing and pre-emptive isolation in the medical ICU) was the primary independent variable. The monthly number of admitted, previously known MRSA carriers was included in the model as a second independent variable, to adjust for colonization pressure [26,27]. All analyses were performed using STATA 8.0 (STATA Inc., College Station, TX, USA).

## Results

### Screening and methicillin-resistant *Staphylococcus aureus *carriage on admission

From April 2003 through March 2004, 2,369 patients (phase I, *n *= 1,316; phase II, *n *= 1,053) were admitted for longer than 24 hours in both ICUs. The proportion of patients who were screened on admission was 41% in phase I (536/1316) and more than doubled in phase II (920/1,053; 87%). In the latter phase, 450 out of 462 surgical ICU patients (97%) and 470 out of 591 medical ICU patients (80%) were screened. From April 2004 (phase III), compliance with on-admission screening remained high in both units (>95%).

In phase II the monthly prevalence of MRSA carriage at the time of ICU admission varied between 4.2% (9/213 screened patients in February 2004) and 7.8% (17/219 screened patients in January 2004). The overall on-admission prevalence of MRSA was 6.7% (71/1053). Without systematic on-admission screening, 55 previously unknown MRSA carriers would have been missed on admission to both ICUs.

### Turnaround times

TATs were determined for 322 and 510 patients screened on admission in phases I and II, respectively. Detailed time savings for both ICUs together are shown in Table 2. Median time from ICU admission to notification of test results decreased from 87 to 21 hours in the surgical ICU (*P *< 0.001) and from 106 to 23 hours in the medical ICU (*P *< 0.001). Of note, we observed not only a reduction in laboratory TATs but also a decrease in the time from admission to screening by advocating MRSA screening as high-priority action on admission.

During phase II, 245 MRSA-negative surgical ICU patients would have spent 1227 additional days in pre-emptive isolation if the culture-based screening technique had been used for systematic on-admission screening. Thus, after implementation of systematic, pre-emptive isolation in this unit, a substantial amount of unnecessary isolation days was saved by using the qMRSA test.

### Methicillin-resistant *Staphylococcus aureus *infection rates

**Figure 1:**
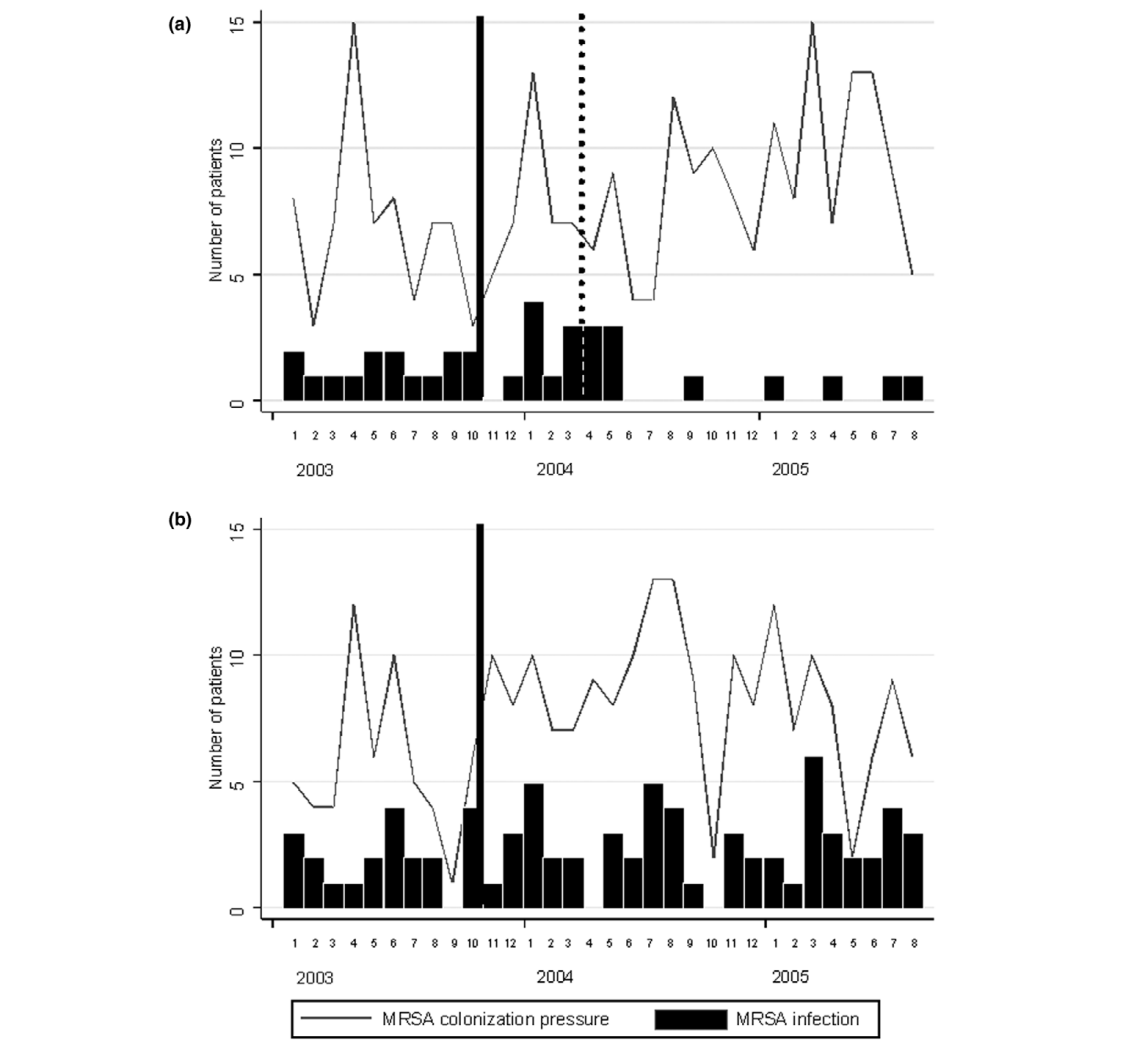
Previously known MRSA carriage on admission versus ICU-acquired MRSA infection. Shown are the numbers of patients with previously known MRSA carriage on admission (MRSA colonization pressure) and the numbers of patients with ICU-acquired MRSA infections (surgical and medical ICUs; Geneva University Hospitals; January 2003 through August 2005). **(a) **(upper panel) Medical ICU. The vertical line on the figure indicates the initiation of rapid on-admission screening in November 2003 (phase II). The dashed vertical line indicates the initiation of pre-emptive isolation for all patients in April 2004 (phase III). **(b) **(lower panel) Surgical ICU. The vertical line on the figure indicates the initiation of rapid MRSA screening on admission and extension of pre-emptive isolation in November 2003 (phase II). ICU, intensive care unit; MRSA, methicillin-resistant *Staphylococcus aureus*.

From January 2003 through August 2005, the overall incidence of ICU-acquired MRSA infections was 1.96 and 4.14 per 1,000 patient days in the medical and surgical ICUs, respectively. The corresponding rates for phase I were 2.7/1,000 patient days (medical ICU) and 3.66/1,000 patient days (surgical ICU), and for phase II they were 4.52/1,000 patient days (medical ICU) and 4.45/1,000 patient days (surgical ICU). As shown in Figure 1, no clear effect on MRSA infections was observed in both ICUs after introduction of the qMRSA test and an increased frequency of screening on admission (phase II). However, a substantial decrease of MRSA infections was seen in the medical ICU after implementation of pre-emptive isolation measures in April 2004 (phase III).

Thirty-two months of consecutive data were available for the Poisson regression analysis. For the surgical ICU, 10 months were used as the control period, whereas for the medical ICU 15 of the 32 months were before the initiation of pre-emptive isolation and rapid on-admission screening. In the surgical ICU model, MRSA colonization pressure was predictive of the number of MRSA infections per month (relative risk [RR] per one-patient increment 1.1, 95% confidence interval [CI] 1.0–1.2; *P *= 0.02), whereas implementation of the qMRSA test had no effect (RR 1.0, 95% CI 0.6–1.7; *P *= 0.97). The combined intervention was a significant independent covariate in the medical ICU model (RR 0.3, 95% CI 0.1–0.7; *P *= 0.004) after adjusting for colonization pressure, indicating that the systematic on-admission screening and pre-emptive isolation strategy was associated with a reduced rate of medical ICU acquired MRSA infections during phase III.

### Characteristics of methicillin-resistant *Staphylococcus aureus *positive patients during phase II

During phase II (November 2003 through March 2004), we collected additional patient-level information on 106 patients with imported or ICU-acquired MRSA. Only 13/106 (12%) patients had their first MRSA isolate from a clinical culture, whereas 71 (67%) were detected through on-admission screening and 22 (21%) in other screening specimens during the ICU stay or at discharge. The median time interval from admission to the first positive MRSA culture in patients who were MRSA-negative on admission was 5 days (range 2–22 days).

Among the 35 patients who were MRSA-negative at admission screening and acquired MRSA carriage during their ICU-stay, 16 (46%) developed at least one MRSA infection. Overall, 22 of the 106 (21%) MRSA-positive patients acquired a MRSA infection in one of the ICUs.

## Discussion

Rapid MRSA screening tests can have an impact both at the individual and group level because they can improve patient outcomes by permitting early detection of MRSA carriage and rapid contact isolation. The principal findings of this study evaluating a rapid MRSA test in critically ill patients are as follows. ICU admission prevalence of previously unknown MRSA carriers was high. Only a small minority of patients had their first MRSA isolate from a routine clinical culture. The qMRSA test decreased overall time to notification from four days to one day and helped to identify previously unknown MRSA carriage rapidly. No effect on MRSA infection rates was observed in the surgical ICU, although a large number of unnecessary pre-emptive isolation days could be saved in this unit by using the qMRSA test. Finally, a substantial decrease in MRSA infections was seen in the medical ICU after increasing compliance with on-admission screening and linking the rapid test to pre-emptive isolation and cohorting of MRSA patients.

Despite the fact that culture-based MRSA screening techniques have proven cheap and sensitive if samples are collected from several body sites, the time to report the results remains a major issue. Definitive identification and testing results are usually available only 48 to 96 hours after sample collection, a time delay that could allow MRSA cross-transmission if patients are not presumptively placed under contact precautions [19,28]. This may be one of the reasons (apart from low hand hygiene compliance) why the recently published study by Cepeda and colleagues [4] did not identify a significant effect of contact isolation for MRSA carriers identified by conventional methods [29].

This study is the first to report detailed time intervals from patient admission to notification of MRSA test results in critically ill patients. Previous reports on rapid MRSA screening tests only assessed the time of specimen processing, without taking into account transport of specimens and the delay between admission and screening [18,30-32]. Our findings show that factors other than laboratory analysis may have an effect on total time from admission to notification of test results, and that efforts are warranted to reduce these delays. The added value of providing molecular screening capability during nights and weekends remains to be assessed.

The few investigations that specifically evaluated the prevalence of MRSA carriage on ICU admission differ from ours in important ways, and so comparisons are limited. However, our study confirms that the prevalence of previously unknown MRSA carriage at admission to critical care is high in settings with endemic MRSA transmission. Previously reported prevalences of MRSA carriage at ICU admission ranged between 6% and 34% [4,6,9,11,33-36]. In all of these studies, fewer than 50% of MRSA carriers had previously been identified as such, emphasizing the importance of early screening to detect the unknown reservoir of MRSA patients.

Current limitations to routine implementation of PCR-based MRSA screening tests are their high costs, the workload of specimen processing and the lack of trained laboratory technicians. We do not yet have sufficient data to demonstrate the cost-effectiveness of our rapid screening strategy. It is possible that MRSA control in the ICU setting may also be achieved with fewer resources and without rapid screening tools [11]. Nevertheless, the rapid qMRSA test saved a large number of unnecessary isolation days in the surgical ICU and helped to decrease substantially MRSA infections in the medical ICU, making it likely that this intervention saved costs for the hospital. In addition, by saving unnecessary isolation days, we might have increased patient safety because isolation precautions may decrease provider–patient interactions and increase the rate of adverse events [37].

In a recently reported mathematical model it was suggested that a policy of screening newly admitted patients for MRSA coupled with rapid contact isolation could reduce nosocomial MRSA infection [38]. However, despite systematic on-admission screening and pre-emptive contact isolation, the incidence of MRSA infections did not decrease in the surgical ICU. By contrast, the medical ICU had greater success with MRSA control. This contradictory finding may reflect differences in case mix, frequency of patient movements outside the ICU, or compliance with standard precautions and isolation practices [39]. Antibiotic selection pressure is rather low in the surgical ICU, making it unlikely that this factor confounded the study results [40].

Our study has limitations. First, we used a rapid MRSA test that lacks perfect specificity and sensitivity. Therefore, we cannot exclude the possibility that the test artificially increased the number of isolation days needed due to false-positive qMRSA test results. Moreover, there may have been patients with false-negative screening results, in whom MRSA acquisition was erroneously attributed to the ICU. Second, we did not perform discharge screening during the entire study period, making it impossible to compare MRSA acquisition rates during the different study phases. Third, although colonization pressure was appropriately adjusted for, other potential confounding factors (such as antibiotic use, compliance with hand hygiene practices and isolation precautions, case-mix, or staffing levels) were not adjusted for in the analysis. Because these factors have been shown to fluctuate over time in a seasonal manner, the baseline ten-month period may have represented a biased estimate of year-round MRSA infection rates. Therefore, the data analysis provided offers only preliminary evidence regarding the effectiveness of the intervention.

## Conclusion

A molecular MRSA detection assay permits rapid identification of MRSA carriage in critically ill patients. It could help to improve MRSA control strategies, especially if it is linked to systematic on-admission screening and pre-emptive isolation of newly admitted patients. Further controlled studies are necessary to evaluate its sustained impact on MRSA cross-infection.

## Key messages

• 	The prevalence of previously unknown MRSA carriage was high at ICU admission.

• 	Only a small minority of patients had their first MRSA isolate from a clinical culture.

• 	Based on a large sample of prospectively monitored sampling procedures, the rapid PCR assay permitted a significant reduction in TAT to report of screening results from four days to one day (during week days), as compared with culture-based assays.

• 	No effect on MRSA rates was observed in the surgical ICU, although a large number of unnecessary pre-emptive isolation days could be saved using the qMRSA test.

• 	A substantial decrease in MRSA infections was seen in the medical ICU after increasing compliance with on-admission screening and implementing a strategy that linked the rapid test to pre-emptive isolation and cohorting of MRSA patients.

## Abbreviations

CI = confidence interval; ICU = intensive care unit; MRSA = methicillin-resistant *Staphylococcus aureus*; PCR = polymerase chain reaction; qMRSA = quick MRSA screening; RR = relative risk; TAT = turnaround time.

## Competing interests

JS and PF are the developers and patent holders of the rapid MRSA test mentioned in the report. All other authors have declared that they have no competing interests.

## Authors' contributions

SH, JS and DP had the idea for the study and wrote the manuscript. SH and CMA developed the study design, drafted the protocol and coordinated its implementation. CMA, CA and GR collected the data and performed part of the analyses. PF made substantial contributions to the development and validation of the MRSA screening test. JP and BR were involved in study supervision and manuscript preparation. All authors read and approved the submitted version of manuscript.

## Figures and Tables

**Table 1:** Summary of the different phases and interventions during the study period

Period	Surgical ICU	Medical ICU
January 2003 to October 2003 (includes phase I, which started in April 2003)	Screening and pre-emptive isolation of high-risk patients only No discharge screening Conventional culture methods	Screening of high-risk patients only No pre-emptive isolation No discharge screening Conventional culture methods
November 2003 to March 2004 (phase II)	Systematic on-admission and discharge screening policy Extension of pre-emptive isolation to all patients Rapid PCR technique for admission screening	Systematic on-admission and discharge screening policy No pre-emptive isolation Rapid PCR technique for admission screening
April 2004 to August 2005 (phase III)	Systematic on-admission and discharge screening policy General pre-emptive isolation Rapid PCR technique for admission screening	Systematic on-admission and discharge screening policy General pre-emptive isolation Rapid PCR technique for admission screening

Note that contact isolation was implemented for all identified carriers of methicillin-resistant *Staphylococcus aureus *during the entire study period. No antibiotic restriction or hand hygiene education campaigns with systematic feedback were performed. ICU, intensive care unit

**Table 2:** Delay between ICU admission and notification of test results: standard culture versus qMRSA

	Standard culture (phase I)	Rapid qMRSA test (phase II)	*P*
*n*	322	510	-
Time from admission to screening (hours)	13.4 (4.8–21.6)	6.3 (0.6–10.3)	<0.001
Time from screening to arrival in the lab (hours)	3.2 (1.3–12.1)	3.6 (1.1–10.9)	0.82
Time from receipt in the lab to result notification (hours)	71.8 (47.9–94.6)	7.2 (6.3–22.2)	<0.001
Total time (hours)	93.1 (73.2–118.9)	22.2 (16.7–27.6)	<0.001

Note that weekends and public holidays were not included. Values are expressed as median (interquartile range). ICU, intensive care unit; qMRSA, quick methicillin-resistant *Staphylococcus aureus *screening.
